# Conservation‐focused mapping of avian migratory routes using a pan‐European automated telemetry network

**DOI:** 10.1111/cobi.70017

**Published:** 2025-03-24

**Authors:** Lucy Mitchell, Vera Brust, Thiemo Karwinkel, Susanne Åkesson, Dmitry Kishkinev, Gabriel Norevik, Tibor Szep, Anders Hedenström, Sander Lagerveld, Barbara Helm, Heiko Schmaljohann

**Affiliations:** ^1^ Terrestrial Ecology Group, Department of Biology, Faculty of Sciences Ghent University Gent Belgium; ^2^ Naturschutzbund Deutschland (NABU) e.V. Berlin Germany; ^3^ Institute of Biology and Environmental Sciences, School of Mathematics and Science Carl von Ossietzky Universität Oldenburg Oldenburg Germany; ^4^ Department of Biology Lund University Lund Sweden; ^5^ School of Life Sciences Keele University Newcastle‐under‐Lyme UK; ^6^ Institute of Environmental Sciences University of Nyíregyháza Nyíregyháza Hungary; ^7^ MME/BirdLife Hungary Nyíregyháza Hungary; ^8^ Wageningen Marine Research Wageningen University and Research Den Helder The Netherlands; ^9^ Bird Migration Unit Swiss Ornithological Institute Sempach Switzerland

**Keywords:** automated radio tracking, demographic parameters, flyway conservation, management plans, migration, Conservación de rutas migratorias, migración, parámetros demográficos, planes de gestión, radio telemetría automatizada

## Abstract

Accelerated biodiversity loss has destabilized functional links within and between ecosystems. Species that cross different ecosystems during migration between breeding and nonbreeding sites are particularly sensitive to global change because they are exposed to various, often ecosystem‐specific, threats. Because these threats have lethal and nonlethal effects on populations, many migratory species are declining, making this group especially vulnerable to global change. To mitigate their decline, research at a continental and flyway scale is required to adequately monitor changes in the migratory and demographic processes of populations during all parts of the annual cycle. The Motus Wildlife Tracking System (Motus) could provide a solution to data gaps that exist for small, migratory species. Motus is an automated telemetry system for animal tracking that uses a single very‐high‐frequency radio signal to track tagged individuals. Motus can provide information on movements made by individuals of small migrant species, thereby aiding the understanding of aspects of their migration that could affect demographic parameters. Conservation‐focused research opportunities related to Motus include identification of critical stopover sites that support and connect multiple species and insight into migratory decisions in small migrant birds related to environmental stressors, such as artificial light at night. Examples of stopover studies from the existing network that demonstrate its utility include identification of a high‐conservation‐value stopover area for the blackpoll warbler (*Setophaga striata*) in the eastern United States. Geographical gaps in the network across the Mediterranean region and across eastern Europe need to be filled to track continent‐wide movements. Motus can provide individual‐level migration information for a variety of small‐bodied taxa, and a drive to expand the network will improve its ability to direct conservation plans for such species.

## Abstract (es)

Cartografía centrada en la conservación de las rutas migratorias de las aves mediante una red paneuropea de telemetría automatizada

**Resumen:**

La pérdida acelerada de biodiversidad ha desestabilizado los vínculos funcionales dentro de los ecosistemas y entre ellos. Las especies que atraviesan distintos ecosistemas durante la migración entre lugares de reproducción y de no reproducción son especialmente sensibles al cambio global porque están expuestas a diversas amenazas, a menudo específicas de cada ecosistema. Dado que estas amenazas tienen efectos letales y no letales sobre las poblaciones, muchas especies migratorias están disminuyendo, lo que hace a este grupo especialmente vulnerable al cambio global. Para mitigar su declinación, es necesario investigar a escala continental y de corredores aéreos para controlar adecuadamente los cambios en los procesos migratorios y demográficos de las poblaciones durante todas las partes del ciclo anual. El Sistema Motus de Seguimiento de la Fauna Silvestre (Motus) podría aportar una solución a la carencia de datos que existen sobre especies pequeñas y migratorias. Motus es un sistema automatizado de telemetría para el seguimiento de animales en el que una única frecuencia de radio VHF rastrea a los individuos marcados. Motus puede proporcionar información sobre los movimientos realizados por individuos de pequeñas especies migratorias, ayudando así a comprender aspectos de su migración que podrían afectar a parámetros demográficos. Las oportunidades de investigación centradas en la conservación y relacionadas con Motus incluyen la identificación de lugares de parada críticos que apoyan y conectan a múltiples especies y la comprensión de las decisiones migratorias en pequeñas aves migratorias relacionadas con factores de estrés ambiental, como la luz artificial por la noche…. Entre los ejemplos de estudios de escalas de la red existente que demuestran su utilidad se incluye la identificación de un área de escala de alto valor de conservación para el chipe cabeza negra (*Setophaga striata*) en el este de Estados Unidos. Es necesario llenar los vacíos geográficos de la red en la región mediterránea y en Europa oriental para seguir los movimientos en todo el continente. Motus puede proporcionar información sobre la migración a nivel individual de diversos taxones de pequeño tamaño, y el impulso a la ampliación de la red mejorará su capacidad para orientar los planes de conservación de tales especies.

## INTRODUCTION

Biodiversity loss driven by land‐use change and exploitation of natural resources and affected further by climatic disruption is a defining feature of the Anthropocene (Sala et al., 2000). A decline in habitat availability and disruption to ecosystem structure, reducing critical services, such as nutrient cycling, carbon storage, and flood control, have led to declines in a wide range of taxa globally (Jaureguiberry et al., 2022). The impacts of anthropogenic development not only manifest through physical changes (i.e., habitat loss) but also through increases in zoonotic and vector‐borne diseases (Jaureguiberry et al., 2022) and pest outbreaks (Ayres & Lombardero, 2018). These impacts affect species’ distributions, abundances, and fitness and consequently their ability to complete their life cycle successfully (Bellard et al., 2012).

Of particular concern are migratory species, which serve as ecological indicators and providers of vital contributions to ecosystem functioning, including biomass production, pollination, and pest control (Bauer & Hoye, 2014; Satterfield et al., 2020). Migratory species experience a variety of environmental conditions on their seasonal, sometimes intercontinental, journeys (Horton et al., 2020; Howard et al., 2020; Turbek et al., 2018; Zurell et al., 2018). Rapid changes in land use and configuration occurring within their annual cycle may mean that their requirements for reproduction and survival are compromised (Birnie‐Gauvin et al., 2020; Marcacci et al., 2022; Rigal et al., 2023). There are also additional threats, such as hunting (Jiguet et al., 2019), modification of physical barriers due to the addition of anthropogenic structures (Gauld et al., 2022), and increasingly unpredictable climatic patterns decoupling the phenology of ecologically linked species (Clarke et al., 2022; Iler et al., 2021).

These challenges directly conflict with the multifactorial optimization of migration, which is often based on inherited, integrated migration strategies (Åkesson & Helm, 2020; Fattorini et al., 2023; Schmaljohann et al., 2022). Although migrant species differ in their migratory timing, distance, speed, and route, their journeys all involve repeated, alternating migratory endurance flights and stopover periods for resting, recovering, and refueling (Åkesson & Hedenström, 2007; Alerstam et al., 2003; Schmaljohann et al., 2022). Understanding the factors affecting population trends of these species (i.e., the changes in vital rates that drive population growth or decline) is essential (Morrison et al., 2016) because many migratory species cannot respond to changes at a sufficiently rapid pace, which can lead to widespread population declines (Both et al., 2006; Frick et al., 2020; Vickery et al., 2023; Wilcover & Wikelski, 2008).

The Convention on the Conservation of Migratory Species highlights the need for a multispecies, flyway‐level perspective in terms of research into population declines (Chowdury et al., 2023; Frick et al., 2020; Marcacci et al., 2022; UNEP, 2020; Vickery et al., 2023). However, gathering data from a sufficiently high number of individuals from different populations at this scale is extremely challenging (McKinnon & Love, 2018; Morrison et al., 2016) and relies on international collaboration (Nadal et al., 2020; Serratosa et al., 2024; Vickery et al., 2023). Particularly for small and light migratory passerines, waders, and highly aerial species such as swifts, their size and behavior make studying their movements difficult (Fiedler, 2009; Wikelski et al., 2007).

## CURRENT METHODS AND THEIR LIMITATIONS

Studying when and where differences in population processes occur in migratory birds is notoriously difficult (Border et al., 2017; Doerr & Doerr, 2005). However, quantifying variation in survival, mortality, emigration, and immigration (summarized as dispersal) is crucially important to formulating effective conservation measures for populations and species that are at risk of decline (DeMars et al., 2023; Gómez et al., 2021).

There is little detailed spatial and temporal information on small birds that migrate. Broadscale migration patterns across Europe, including concentrations of avian and insect migrants passing through marine and mountainous regions, have been identified using radar (Bruderer & Jenni, 1990; Bruderer & Liechti, 1999; Hirschhofer et al., 2024; Nilsson et al., 2019; Weisshaupt et al., 2021). Yet, radar data largely do not allow researchers to tease out species‐specific and individual‐level variation in large‐scale movements (Schmaljohann et al., 2008; Zaugg et al., 2008). Such information would facilitate linking individual migratory behaviors to demography, physiology, and ecology.

Several million individuals have been marked with metal or color rings across Europe (Du Feu et al., 2016; Spina et al., 2022), contributing to fundamental knowledge of bird movements. Yet, recapture, recovery, and resighting probabilities are relatively low (across 32 European‐level ringing schemes, recovery rate for all species combined ranges from 0.6% to 7.6% [Baillie, 1995]). This is particularly the case on wintering grounds but is highly variable among species and locations (Thorup et al., 2014). For example, the willow warbler (*Phylloscopus trochilus*) is ringed in huge numbers on its breeding ground in northern Europe, but only a very small number of recoveries occur in Africa. For each recovery of this species in sub‐Saharan Africa, 16,000 individuals need to be marked on the breeding grounds in Finland (Hedenström & Pettersson, 1987).

**FIGURE 1:**
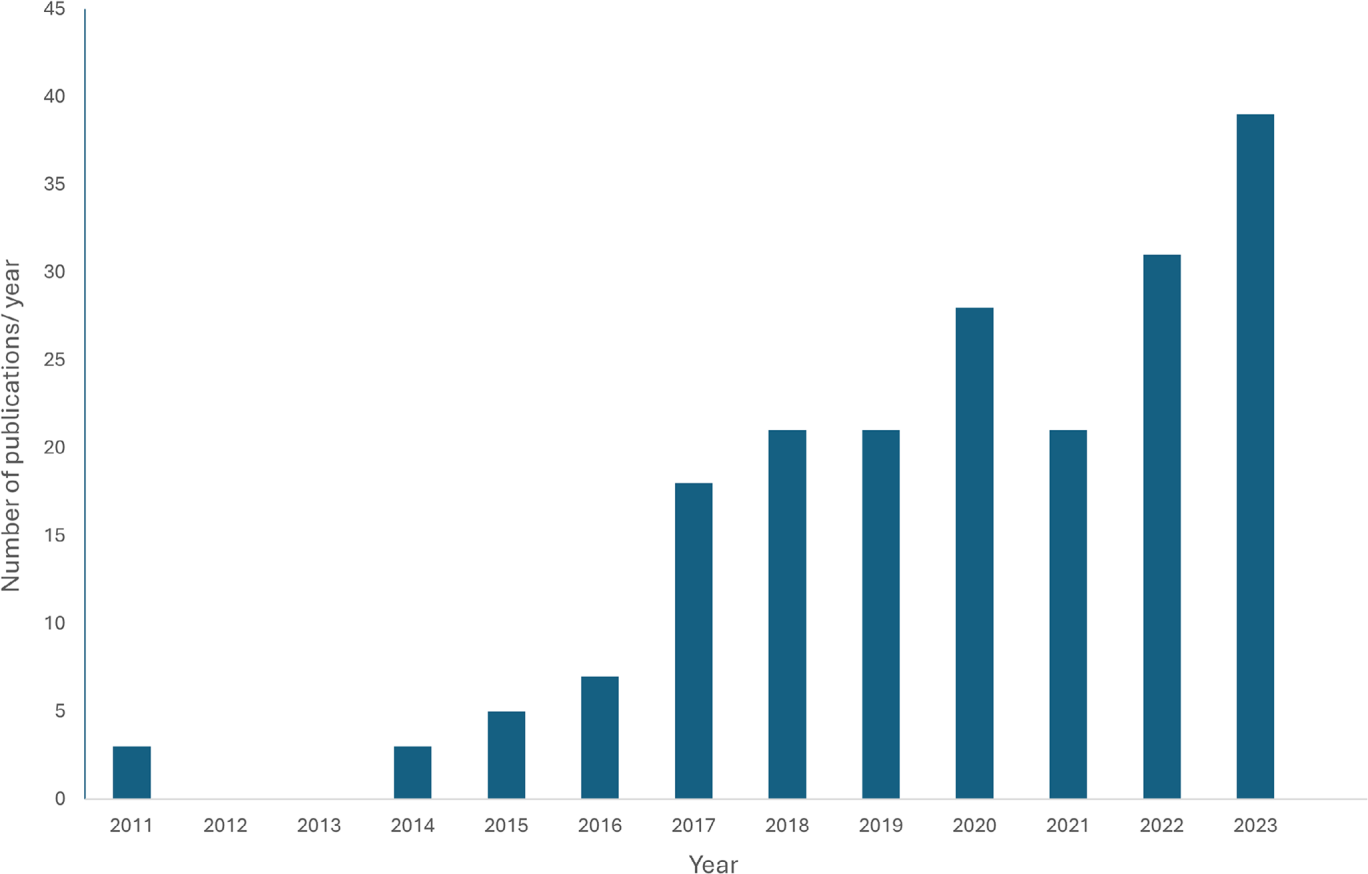
Number of publications per year resulting from Motus data (*Source*: motus.org).

The disadvantages of these methods can be mitigated by tracking individual migratory prebreeding and postbreeding dispersal (Mukhin et al., 2005; Züst et al., 2023) and nomadic, nonbreeding movements (McKinnon et al., 2019; Snell et al., 2018). However, individual tracking of small migrants requires tracking devices weighing a maximum of 3%–5% of an individual's body weight (Barron et al., 2010), which excludes most tracking technology on the market (Figure 1) (Bridge et al., 2011; McKinnon & Love, 2018), including new low‐power, wide‐area devices, such as SigFox and LoRaWAN (Wild et al., 2023). Radio transmitters, however, have already reached minimum weights of currently 0.13 g (Lotek NanoPin), lighter than the smallest light‐level geolocators (Lotek: 0.3 g) and significantly smaller than GPS units that require significant energy to relay information to a satellite and fix a position. Some radio‐tracking systems (e.g., tRackIT System and ATLAS project) have narrow spatial coverage and a limit to how many individuals (ca. 200) can be monitored concurrently (Beardsworth et al., 2022; Gottwald et al., 2019).

The Motus Wildlife Tracking System (Motus) exploits a network of very‐high‐frequency (VHF) receiving stations equipped with directional Yagi antennae on the same frequency. These receivers continuously receive and record uniquely coded signals from tagged individuals, and there is no need for recapture (Imlay et al., 2020; Taylor et al., 2017). We focused on how Motus can fill conservation and demographic‐specific knowledge gaps through tracking of migratory birds. We sought to spark further collaborative use of Motus to create a denser network in Europe that resembles the situation in North America.

## MOTUS AND ITS BENEFITS

Motus was devised through a partnership between Acadia University and Birds Canada researchers (Taylor et al., 2017, 2011), and its spread across the Americas is a great success story of collaborative research (https://motus.org). Globally, to date (December 2024) there are 899 tagging projects, which combined have tagged 51,089 animals of 403 species. The entire Motus network consists of 2066 receivers, and the largest single project array consists of 109 receivers in Ontario, Canada.

**FIGURE 2:**
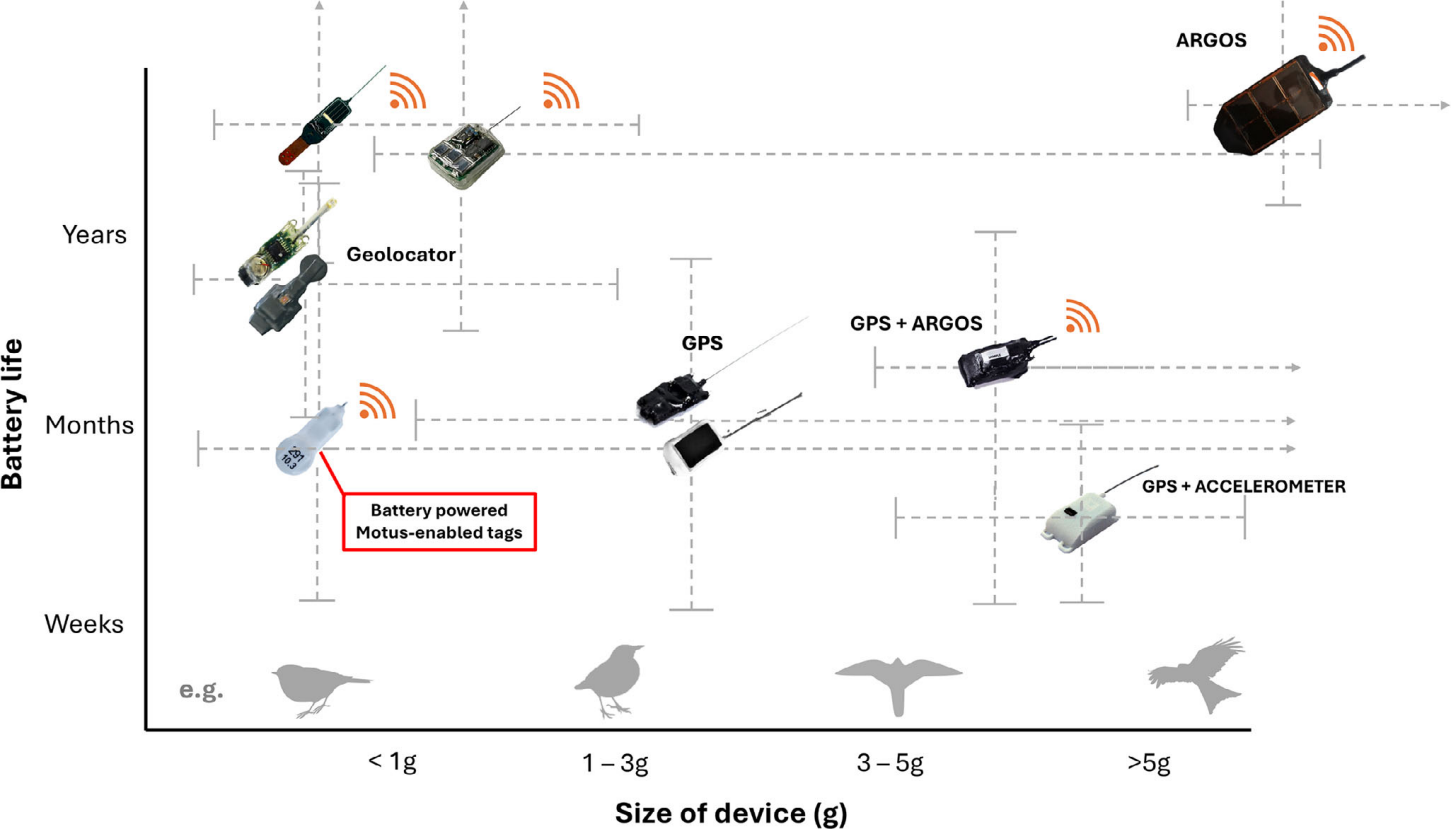
Capability and context of tags enabled for Motus (gray dotted lines, variation on both axes taking into account programing influence on battery life and differences among and between device types; orange Wi‐Fi symbols, transmission capability independent of bird's return to a specific location). Tag types are positioned approximately in relation to their mean battery lifetime and size.

Publications resulting from Motus data total 218, which combined were cited, according to Zotero, >500 times. The number of publications based on Motus data has more than doubled since 2015 (Figure 2), and the lead and coauthors of these publications are rarely limited to academics. The application of Motus has been recognized by multiple stakeholders, such as the US Fish and Wildlife Service, National Parks Service, Canadian Wildlife Service, and BirdLife Europe (Machado et al., 2024).

Investment in the network across North America continues to grow. In March 2024, a grant of CSD3.1 million was awarded to a consortium of 5 Canadian universities and Birds Canada to continue installing Motus receiving stations across the country and to further community‐based science. This investment, combined with a specific mention in the Convention on Migratory Species of automated radio tracking deployed at a flyway scale (COP13, Resolution 12.26), demonstrates the current and potential future value of Motus to conservation.

**FIGURE 3:**
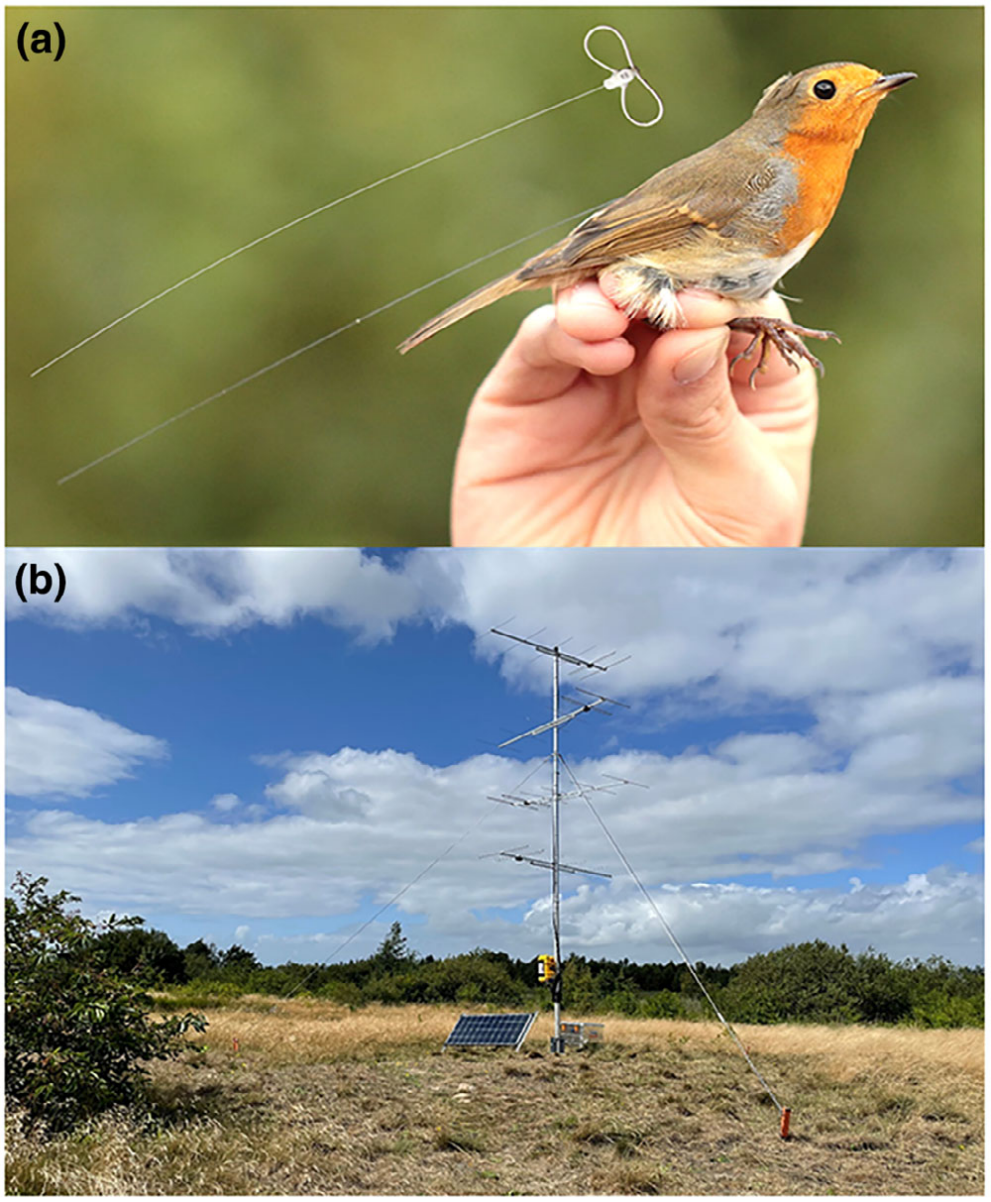
(a) European robin (*Erithacus rubecula*) with attached radio transmitter and leg‐loop harness (shown above the bird) and (b) a Motus receiving station (6‐m tall) with 4 six‐element Yagi antennas pointing in 4 directions. The station is solar powered and has a buffer battery (in aluminum box on ground). Electronics are in the yellow box on the pole. Detailed information about tagging animals and building stations is on the Motus Webpage (motus.org/resources/) and available from regional Motus coordinators (motus.org/groups/regional‐coordination‐groups/). Photos by T.K.

**FIGURE 4:**
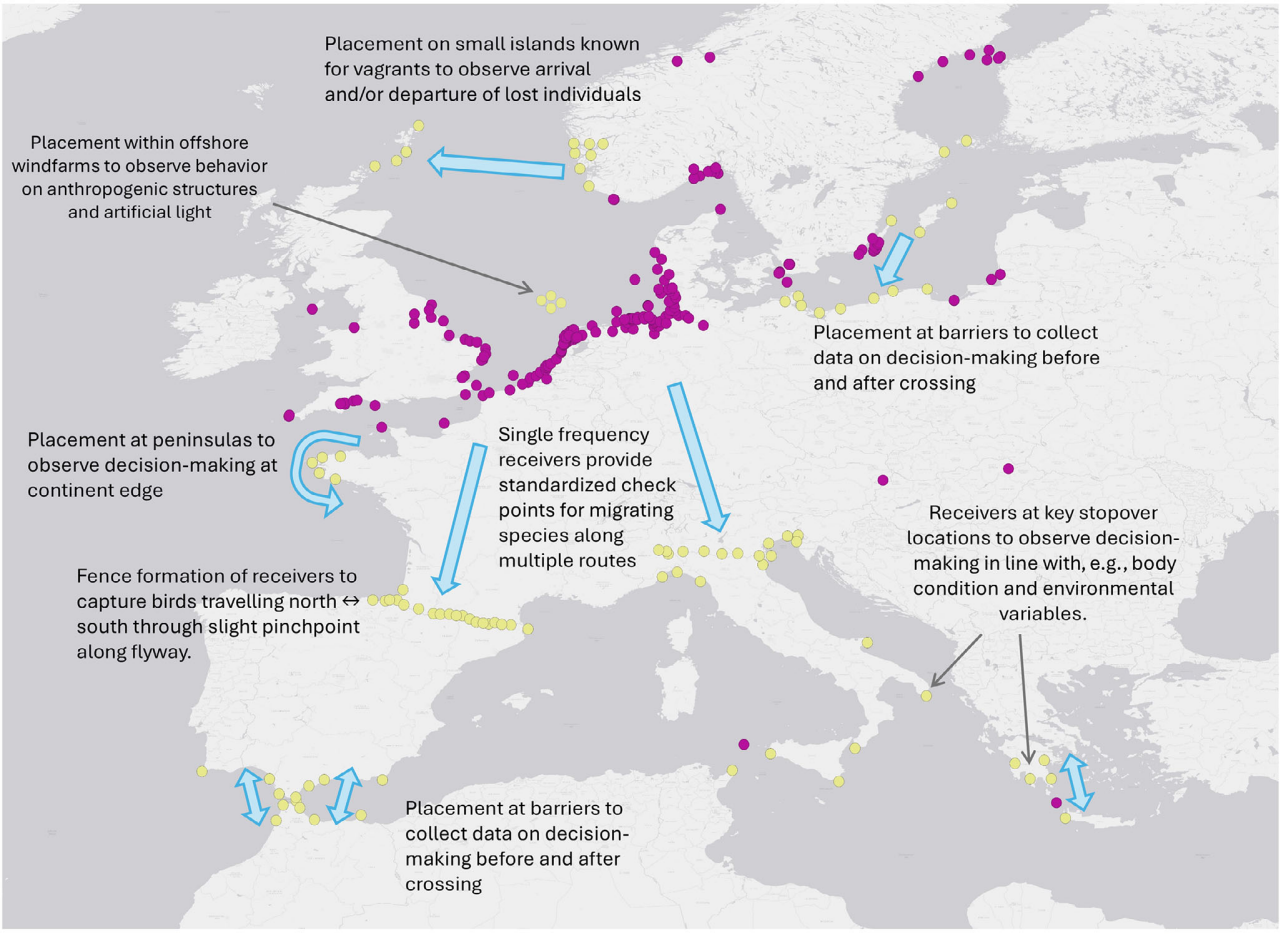
Current Motus receiving station network (purple dots) across Europe and hypothetical future stations (yellow dots) to show the potential Motus data have to answer demographic and conservation‐focused questions about bird migration (blue arrows, flyways and movements of interest).

The initiation of Motus in Europe started in 2017. Although the network has grown more slowly than in the Americas, there is now a dense network of passive receiving stations (Figure 3) along the coasts of Germany, the Netherlands, and the United Kingdom and to a slightly lesser extent in Sweden, Denmark, Belgium, and France. There are a number of stations in other countries as well as offshore (Figure 4).

The network of stations in Europe is patchy, particularly in eastern Europe, and there is a lack of a universally permitted tracking frequency, so it does not yet allow continuous tracking across the continent. In many European countries, the frequency of 150.1 MHz is authorized temporarily or permanently for wild animal telemetry tracking. Multifrequency detection by Motus receivers is possible, but it incurs additional expense for extra equipment. For example, adding antennas to receiving stations for monitoring the license‐free frequency of 434 MHz would cost approximately €80–300 per station, for an additional 1–4 antennas, plus the cost of extra cables. For the tags, researchers can select from among a number of options and device parameters, including burst interval (usually from 1 s to 1 min), battery or solar power, attachment type, and antenna type, to meet their specific scientific requirements (Figure 1).

Motus has many promising features, including its extended temporal and spatial data gathering capacity, relative to standard radio tracking. In addition to autonomous, near‐real‐time recording of the receivers and sub‐0.5‐g tags, the spatial scale of detections is in the order of several kilometers, rather than orders of magnitude higher, as with geolocators (Taylor et al., 2017), although new multisensor tags have improved substantially in positional accuracy (Nussbaumer et al., 2023). Receiving stations can, in theory, be placed anywhere (Figure 3b) and have a detection range of >10 km; therefore, data capture is less limited by researcher effort, in contrast to commonly used methods, such as bird ringing (Flack et al., 2022; Griffin et al., 2020). Fixed positioning of the receiving stations (ideally at sites of importance to the species of interest to maximize detection probability) and an unrestricted recording period allow standardized data collection and thus reduce observer bias (Griffin et al., 2020).

Despite the advantages, there are drawbacks of radio‐tracking studies in general. Most studies do not detect all tagged individuals. The reasons for this are many and not mutually exclusive but include loss of the tag, predation, emigration, tag failure, topography, and weather conditions. Crewe et al. (2020) reported detection rates of 50%–70%, whereas a dense coverage of receivers on the small island of Helgoland, Germany, resulted repeatedly in detection rates of 95%–100% (Karwinkel et al., 2024, 2022). There are also uncommon occurrences of high levels of false positive, or uncoded detections, which can appear if large numbers of individuals are released at once close to a receiver. Mitigations, such as staggered switching on of the tags to encourage differential pulse emission, can be put in place, and the numbers required to cause this confusion mean this is unlikely to happen in a natural scenario.

Motus is already producing important insights into the movements of migrating and wintering European birds, including a better understanding of the migratory and premigratory movements of sea‐crossing thrushes (Brust et al., 2019) and differences between long‐ and short‐distance migrants in stopover time and flight direction (Packmor et al., 2020; Rüppel et al., 2023). Examples from Europe and North America show that Motus can gather long‐term, annual cycle data, for a relatively low cost, on groups and in periods (e.g., juvenile fledging) that are often missing from population studies (Martell et al., 2023; Satterfield et al., 2020).

## HOW MOTUS CAN HELP TO ADDRESS KNOWLEDGE GAPS IN SMALL MIGRATORY BIRD DEMOGRAPHICS

### Survival and mortality

Despite the biological significance of survival and mortality to population size and its dynamics (Sandercock et al., 2020), little is known about either rate in migratory passerines. In migratory species, variation in survival among populations can be linked to alternative routes and their different pressures (Hewson et al., 2016). The latter may increase population‐specific immediate and delayed fitness costs (Dhanjal‐Adams et al., 2017), which might be particularly prevalent in areas that support high numbers of comigrants (multiple species moving through major sites and corridors simultaneously [Cohen & Satterfield, 2020]). The convergence of otherwise spatially segregated populations at single locations may also increase the probability of disease transmission, which can have delayed fitness costs (Cohen & Satterfield, 2020).

To obtain information on route‐ or area‐specific mortality rates, focusing receiving station placement in closely packed fence or curtain formation (Figure 4) would provide checkpoints for tagged migrants along their migratory routes. If there are sufficient stations intersecting migratory routes (and adequate numbers of individuals are tagged), then obstacles that slow down migration can be identified and estimates of mortality rates for such areas can be made (Buechley et al., 2021; Klaassen et al., 2014). Survival has been estimated successfully based on Motus data for Kirtland's warbler (*Setophaga kirtlandii*) (Cooper et al., 2024). This species, with its limited population size and discrete wintering range, lends itself to Motus tagging, and a robust design Cormack–Jolly–Seber model allowed the calculation of apparent survival rates with a high level of certainty, knowing that a high proportion of marked individuals had been detected.

Gonzalez et al. (2021) used Motus data to identify habitat‐specific overwinter survival rates in Swainson's thrush (*Catharus ustulatus*), which can be used to inform habitat protection and management on the wintering grounds. Motus data have also been used by Brunner et al. (2022) to identify high migratory connectivity among populations of the elusive Swainson's warbler (*Limnothlypis swainsonii*), which has implications for population‐specific changes and can direct future monitoring work. These cross‐continental studies demonstrate the power of Motus to collect data at multiple scales along the length of a flyway. Extensive testing of detection capability of an antenna array in a fixed area is essential to maximizing coverage and the ability to produce survival estimates. It is better yet if survival is estimated across a limited area (Cooper et al., 2024) and a restricted temporal period to increase the robustness of the estimates (Evans et al., 2020).

### Identification of stopover sites

Motus can be used in regional arrays that expand outward from a known stopover site, allowing identification of exploratory and regional movements by birds that may be assessing the wider area, often at night (Brown & Taylor, 2015; Schmaljohann & Eikenaar, 2017; Taylor et al., 2011). In Europe, this could build on current ringing efforts at hotspots of bird occurrence (e.g., Bay of Biscay, Strait of Gibraltar) at spatial scales not feasible with ringing alone. Pinpointing specific sites for targeted conservation efforts is important; limited, localized stopover site use can increase vulnerability in certain migrating species (Bayly et al., 2013; Gómez et al., 2014; Hagelin et al., 2021).

Information on arrival and departure times from Motus data on multiple individuals of different species can help elucidate the functions of stopover sites (Linscott & Senner, 2021; Moore, 2018; Schmaljohann et al., 2022). Identification of these functions could be very valuable in the context of future global climate change, when the current conditions of stopover sites may degrade or even disappear (Bayly et al., 2018). Smetzer and King (2018) used data from a regional Motus network to identify a major stopover area for blackpoll warblers (*Setophaga striata*) and red‐eyed vireos (*Vireo olivaceous*) in the Gulf of Maine of the United States. The prolonged stopovers recorded for both species suggest that the region may be a major refueling area for birds preparing for very long‐distance migratory flights, thus demonstrating the area's high conservation value.

Stopover sites on either side of ecological barriers could be equipped with Motus stations at high densities (e.g., 5–10 km between stations, but variation in detection distance due to topography and the behavior of the species must be taken into account) to provide insights into how birds respond to such barriers (e.g., Sjöberg et al., 2015; Zenzal et al., 2021). This might include local‐ to regional‐scale movements before crossing a barrier, intrinsic and extrinsic conditions required for a successful crossing, stopover duration, departure directions, and potential differences between populations and seasons. Holberton et al. (2019) and Herbert et al. (2022) used Motus data to demonstrate site‐based variation in stopover duration, which was related, at least in part, to bird condition and morphology. This indicates some level of migratory connectivity and thus that loss or degradation of one or more stopover sites could have population‐level effects.

### Dispersal, immigration, and emigration

Natal and breeding dispersal are critical but understudied fundamental biological processes, partly because the survival rates of nestlings and juveniles are generally so low that high personnel and financial investments are required to track a few individuals. Dispersal consists of the initial process of emigration from a breeding site and the subsequent immigration to another (Matthysen & Clobert, 2012).

Species with discrete breeding sites restricted by habitat may become increasingly inbred, and this inbreeding could be exacerbated by habitat loss, climate change, and a lack of immigration (Day et al., 2024). Such changes may consequently lead to a species’ rapid decline if survival is also low (Schaub et al., 2013, 2012). Understanding how these populations are connected through immigration and emigration (e.g., as in le Roux & Nocera [2021] who used Motus data on chimney swifts [*Chaetura pelagica*]) is important for deciding what conservation measures might be useful to avoid loss of genetic diversity (Driscoll et al., 2014). Researchers can estimate emigration and immigration rates of a species of interest through comprehensive tagging campaigns (ethical considerations of such projects notwithstanding [Soulsbury et al., 2020]), where Motus stations can cover initial breeding sites and potential areas where birds might disperse.

Regional‐scale movements of juvenile blackpoll warblers, Kirtland's warbler, and barn swallow (*Hirundo rustica*) have been demonstrated prior to migration based on data from the Motus network (Brown & Taylor, 2015, 2017; Cooper & Marra, 2020; Evans, 2018). Data are particularly needed from juveniles to assess when, how, and why they decide on breeding site settlement. Studies by Doerr and Doerr (2005) and Mukhin et al. (2018) suggest that tracking the dispersal of breeders and fledging juveniles to new habitats in the region is feasible using this system.

Questions remain about the function of exploratory dispersal movements, which may be preparatory information‐gathering trips (“homing target” or “habitat optimization” hypotheses [Mitchell et al., 2015]), and premigratory flights (Züst et al., 2023). These flights may relate to range expansion and individual or species responses to climate change (Driscoll et al., 2014; Dufour et al., 2022, 2021). Tracking individuals during the dispersal phase could improve understanding of the role of (long‐distance) dispersal in the evolution of new migration routes and wintering grounds, perhaps as part of the wider phenomenon of vagrancy (Dufour et al., 2022, 2021; Lees & Gilroy, 2009).

Motus’ ability to expand spatially and temporally beyond the capabilities of manual VHF tracking, thus reducing bias and monitoring hidden movements (Züst et al., 2023), can then increase the power of studies on juvenile fledging movement (Cox & Kesler, 2012) and medium‐ to long‐distance postbreeding dispersal (Evans et al., 2018; Hayes et al., 2024). Results from such studies can inform conservation decisions and improve understanding of how far and in what direction juveniles disperse. Tracking of many different young individuals can also highlight how individual phenotypes and differences in body condition might lead to differential postfledging survival (Motus data used in a study of fledging barn swallows [Evans et al., 2020]) and how this might be affected by surrounding habitat quality (wood thrush [*Hylocichla mustelina*] [Hayes et al., 2024]). These practical elements are invaluable to formulating effective conservation measures and facilitating population stability (Endriss et al., 2019; Niebuhr et al., 2015; Travis & Dytham, 2013).

### Understanding migratory decisions

In addition to using Motus data to describe migration, it can facilitate an experimental approach (i.e., extending laboratory‐based studies in natural scenarios) (Goymann et al., 2010; Schmaljohann & Klinner, 2020). For instance, by radio tagging multiple lean and fat individuals of a species on a single day, researchers can minimize the effect of weather variation on the birds’ departure decision and separate out phenotypic, condition‐related variation (e.g., Karwinkel et al., 2024, 2022). Subjecting numerous individuals to the same external conditions and tracking them at the same time may allow estimation of conditions when most individuals migrate (Delingat et al., 2008; Schmaljohann & Klinner, 2020), for example, during favorable winds (Lagerveld et al., 2024).

**FIGURE 5:**
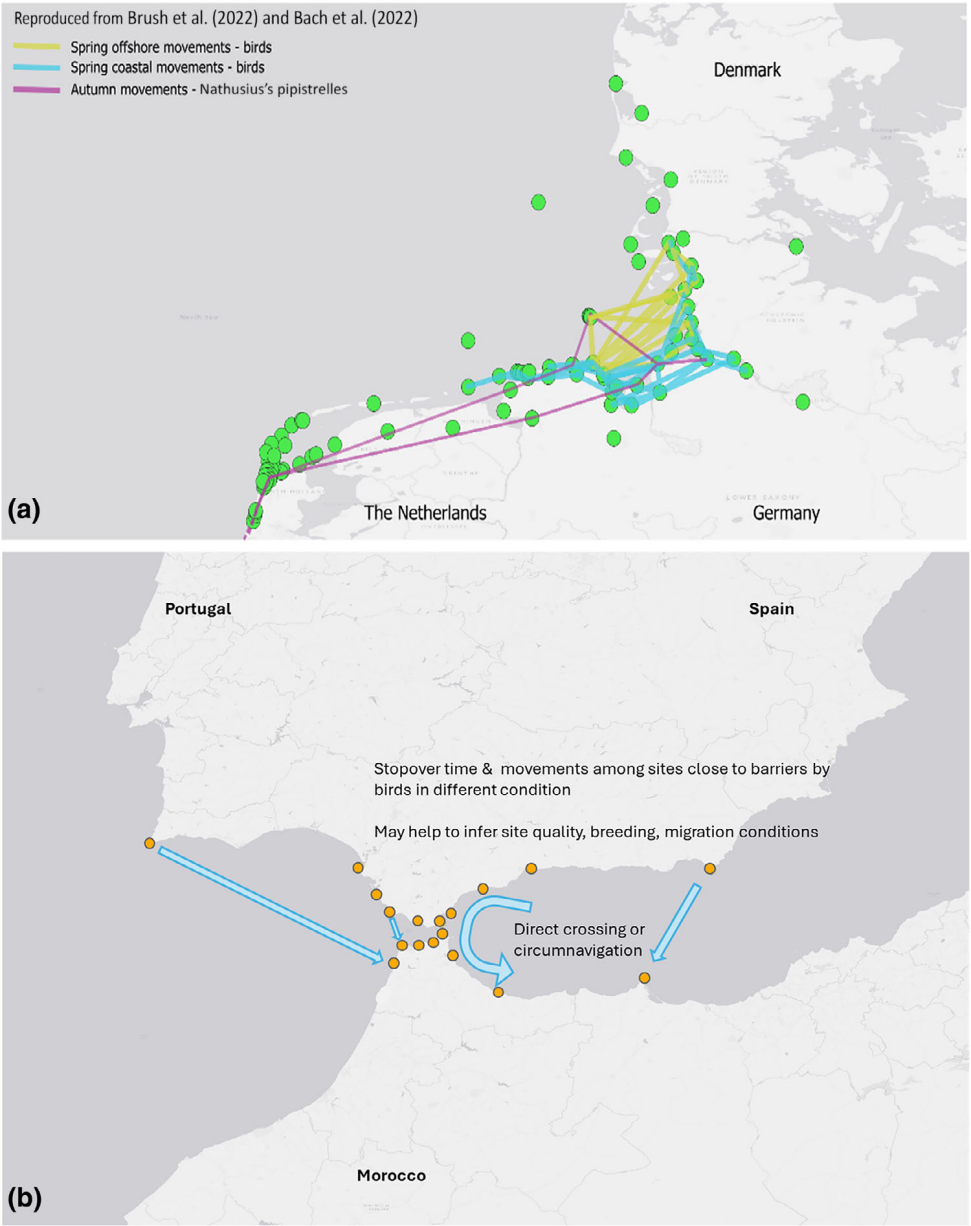
(a) Currently operational Motus receiving stations (green dots) along the North Sea coast and examples of movement tracks of birds and bats and (b) examples of potential station placement (yellow dots) and data collection at Gibraltar, Iberian Peninsula, where many thousands of migratory species cross an important migratory barrier, the Mediterranean Sea (blue arrows, expected flight paths that could be detected by Motus).

Parameters derived from flights of individuals tracked with Motus, such as departure and landing decisions, speed, and routes (Figure 5a; Brunner et al., 2022; Brust et al., 2019; Linhart et al., 2023; Packmor et al., 2020; Ruppel et al., 2023), can allow comparisons in behavior among individuals of different populations and those that orient across and around barriers (Figure 5b) (Brust & Hüppop, 2022; Schmaljohann & Naef‐Daenzer, 2011; Woodworth et al., 2015). An improved understanding of migration behavior, its limitations, and flexibility among different species can help improve predictions of how species might adapt to changes around them and thus improve efforts toward their conservation (Sutherland, 1998).

### Obtaining individual responses to environmental stressors

Motus data can also be used to identify issues of conservation concern and detect responses of migrating birds to specific forms of anthropogenic or environmental disruption. Anthropogenic structures, such as offshore wind turbines, can attract migrating birds, potentially causing increased mortality through collision (Perrow, 2019) or evoking avoidance behavior that can lead to increased or delayed fitness costs due to longer routes and higher energy expenditure (Schwemmer et al., 2023). Such impacts are still largely unquantified for migratory populations of birds (Marques et al., 2021). One possibility is to use Motus in combination with acoustic monitoring (as in Lagerveld et al. [2023]), whereby it is possible to localize the interaction of tracked individuals with near‐ and offshore infrastructure while also contextualizing these known individuals among con‐ and allospecifics detected by the acoustic recorders (Loring et al., 2019; Willmott et al., 2023).

Other anthropogenic disruptors are agrochemicals, such as neonicotinoids, which can impair the progress of migration in different taxa (Cabrera‐Cruz et al., 2020). Eng et al. (2019) used Motus tracking data to show responses to neonicotinoid ingestion by white‐crowned sparrows (*Zonotrichia leucophrys*). They showed that migrating birds on stopover were severely impaired in their ability to refuel, despite significantly increasing the length of stopover.

Artificial light at night (ALAN) can attract night‐migrating birds to bright, often urban, areas (Horton et al., 2023; McLaren et al., 2018; Smith et al., 2021). These areas may act as ecological traps (i.e., inadequate stopover sites that might present higher risk of mortality [Van Doren et al., 2021]). Similarly, anthropogenic electromagnetic radiation (electrosmog) may disrupt the magnetic compass of night‐migrating songbirds (Engels et al., 2014). Engels et al. observed these responses in a laboratory environment with caged birds; whether electrosmog is also a hazard for freely moving birds in the wild remains to be tested. Here, researchers could apply Motus tracking, where directional and departure time data can be collected by local and regional arrays of receivers positioned in and around areas of high urban density.

### Combining Motus tracking with physical samples

Simultaneously collecting samples (e.g., feathers, saliva, blood, or feces) that reveal something about the physiological state of the animals, together with movement behavior, can improve understanding of how the physiology of an individual influences its migratory decisions. The high temporal resolution of Motus tracking data allows one to identify more closely links between physiological indicators, especially those changing rapidly (e.g., hormones), and species’ movement (e.g., Eikenaar et al., 2020). This could, for example, include site quality by correlating stopover duration and habitat use, as recorded by Motus, with body condition and immune function (Brust et al., 2022; Hegemann et al., 2018; Schmaljohann & Naef‐Daenzer, 2011). This would allow researchers to determine whether the sites provide the necessary functions for stopover. If not, targeted conservation measures could be taken to restore the missing functions.

Genetic analyses in conjunction with recorded migratory behavior (direction and routes, which are accessible with the high spatiotemporal accuracy of Motus) could indicate population‐specific differences and possibly regions in the genetic structure that are important for the genetic coding of migratory behavior (Bossu et al., 2022; Ruegg et al., 2014; Sharma et al., 2023). Blood and fecal samples could be used to monitor the prevalence of pathogens that can be linked to body condition, population origin, and migration decisions (ideally seasonal migration success) (Morales et al., 2022; Neima et al., 2020). In the long term, standardized studies of migratory behavior combined with sampling of tagged individuals could allow predictions of responses to global climate and habitat changes (Anderson et al., 2019; Saura et al., 2014).

### Logistics of developing Motus for flyway‐level research

Achieving greater geographical (i.e., near‐continental) coverage of the Motus network stations is underway. However, this requires a strategic placement plan (Lefevre & Smith, 2020) based around the key questions we have discussed and the special physical features of European landscapes (Figures 4 & 5). The network will require significant capital investment and a collaborative spirit among researchers, conservationists, and volunteers because this task is too big for single groups.

Single groups can realize regional‐scale networks through discrete projects, which is a necessary way of completing a continent‐wide network (Griffin et al., 2020; Taylor et al., 2017). Ideally, such projects fill in geographical gaps based on species ecology and migratory behavior already garnered from other technologies (e.g., geolocators [Bayly et al., 2018] or radar [Robinson, 2023]). As well as capital, the development of the network will require significant time and focus to maintain equipment and retrieve data, particularly in remote areas. Such receivers are less likely to be monitored remotely because of signal and power restrictions; therefore, greater logistical efforts are required to obtain the stored data and undertake maintenance.

Cost per receiver can be realized for as little as €3000–5000 (∼4 directional antennas, Sensorgnome receiver), but it may approach €10000 depending on requirements for installation and precise configuration of antennas. Each tag, whether from CTT or Lotek, is approximately €200, although this approaches €300 for the very smallest models. Although cheaper than large, satellite‐enabled tags, this does not approach the low cost of metal or color rings that allow researchers to capture and mark many thousands of birds. Cost reduction is hampered by limited market competition and a lack of open‐source development, which contrasts with the collaborative nature of Motus entirely and must be addressed to allow tagging on a much larger scale.

Finally, the amount of data collected from Motus is enormous and is likely to continue to grow alongside other biologging data (López‐López, 2016), so appropriate statistical tools will need to continue to be developed. Complex Bayesian modeling frameworks to appropriately analyze Motus data have been developed and tested in limited circumstances (e.g., modeling movement offshore [Baldwin et al., 2018; Cranmer et al., 2017] and estimating flight heights [Lagerveld et al., 2024]). Extending the applicability of these methods and developing integrated frameworks with multiple data types would allow researchers to make better use of Motus data and make further inferences about migratory parameters that can inform conservation (Gregory et al., 2023).

These challenges can only be solved over the long term with a coordinated, international, collaborative effort to develop joint funding applications and to work together for the benefit of the wider Motus community. This community must contain academics, policy makers, government officials, conservationists, and amateur biologists and ecologists who can develop well‐defined, focused study objectives. The involvement of a diverse number of stakeholders is required, not just to share the cost burden and coordination responsibilities but also to ensure fair data sharing and the direct integration of such data into policy and conservation actions (Gregory et al., 2023; Guilherme et al., 2023; UNEP, 2020).

## FINAL OUTLOOK

In this time of rapid ecosystem disruption, it is vital to work collaboratively to conserve migratory species. Work needs to be done at multiple scales to answer questions about how species are confronting environmental changes. Motus can provide multiscale data on the movements of bird without the need for recapture, even on species as small as Nathusius's pipistrelles (*Pipistrellus nathusii*) (Bach et al., 2022; Briggs et al., 2023; Lagerveld et al., 2024), yellow‐browed warbler *Phylloscopus inornatus*), and large insects, such as the monarch butterfly (*Danaus plexippus*) (Knight et al., 2019; Wilcox et al., 2021). Motus’ features and capabilities make it an attractive and exciting prospect for exploring as‐yet‐unanswered ecological, evolutionary, and behavioral questions.

There is a significant amount of logistical and planning work to be done to develop and increase the size of the network so that it can reach its full potential in terms of basic and applied science. Such efforts should also focus on expanding the collaboration between parties and realizing conservation strategies that will benefit birds, nature as a whole, and ultimately, by supporting the One Health approach, humans.
